# Still feeling it: the time course of emotional recovery from an attentional perspective

**DOI:** 10.3389/fnhum.2013.00201

**Published:** 2013-05-21

**Authors:** Jayne Morriss, Alexander N. W. Taylor, Etienne B. Roesch, Carien M. van Reekum

**Affiliations:** ^1^Centre for Integrative Neuroscience and Neurodynamics, School of Psychology and Clinical Language Sciences, University of ReadingReading, UK; ^2^Centre for Integrative Neuroscience and Neurodynamics, School of Systems Engineering, University of ReadingReading, UK

**Keywords:** emotion, attention, reactivity, recovery, face, late positive potential, P3, N170

## Abstract

Emotional reactivity and the time taken to recover, particularly from negative, stressful, events, are inextricably linked, and both are crucial for maintaining well-being. It is unclear, however, to what extent emotional reactivity during stimulus onset predicts the time course of recovery after stimulus offset. To address this question, 25 participants viewed arousing (negative and positive) and neutral pictures from the International Affective Picture System (IAPS) followed by task-relevant face targets, which were to be gender categorized. Faces were presented early (400–1500 ms) or late (2400–3500 ms) after picture offset to capture the time course of recovery from emotional stimuli. Measures of reaction time (RT), as well as face-locked N170 and P3 components were taken as indicators of the impact of lingering emotion on attentional facilitation or interference. Electrophysiological effects revealed negative and positive images to facilitate face-target processing on the P3 component, regardless of temporal interval. At the individual level, increased reactivity to: (1) negative pictures, quantified as the IAPS picture-locked Late Positive Potential (LPP), predicted larger attentional interference on the face-locked P3 component to faces presented in the late time window after picture offset. (2) Positive pictures, denoted by the LPP, predicted larger facilitation on the face-locked P3 component to faces presented in the earlier time window after picture offset. These results suggest that subsequent processing is still impacted up to 3500 ms after the offset of negative pictures and 1500 ms after the offset of positive pictures for individuals reacting more strongly to these pictures, respectively. Such findings emphasize the importance of individual differences in reactivity when predicting the temporality of emotional recovery. The current experimental model provides a novel basis for future research aiming to identify profiles of adaptive and maladaptive recovery.

## Introduction

Emotional events that bear relevance to an organisms' well-being, demand center stage in selective attention, and initiate a cascade of typical behavioral and psychophysiological response tendencies (Frijda, 1986; Davidson, 1998; Lang and Bradley, 2010). These responses can be considered to originate from an adaptive *emotional auto-regulation* process (Kappas, 2011); that is, modification of the intensity in emotional responding, or termination of emotional responding, is instigated without motivation, in order to avoid negative stimuli, approach positive stimuli, or return to a steady emotional state. Whilst the majority of research in the field examines affective responding upon the onset of an emotional stimulus, few studies have investigated the extent to which emotional responding continues after stimulus offset (Jackson et al., 2003; Hajcak and Olvet, 2008). Examining the time course of auto-regulation after the offset of an emotional event may provide crucial information to understand adaptive *recovery time*, which is described in the *affective chronometry* model, as the speed of return to a baseline state in a response system after an emotion eliciting stimulus (Davidson, 1998). A landmark study by Jackson et al. (2003) tested this notion by collecting electroencephalography (EEG) frontal asymmetry data to serve as a metric of individual differences in affective style, which was then used to predict outcomes on physiological markers of recovery such as eye-blink startle in an emotional task. The task in this study consisted of presenting International Affective Picture System (IAPS) pictures for 6 s, with audio probes presented either 2.5 or 4.5 s during the picture or 1 s after picture offset. Jackson and colleagues found individuals with right electrical frontal-asymmetry to have larger eye-blink startle magnitude to an audio probe presented 1 s after a negative picture, compared to a neutral picture. Furthermore, with a similar paradigm Larson et al. (2007) found healthy participants exhibiting depressive and anxiety symptoms to have different profiles of emotional recovery. For example, those with depressive symptoms had shown a blunted startle response to audio probes presented 1.5 s after positive pictures, compared to controls. In addition, individuals high in anxious apprehension showed potentiated startle to audio probes presented 1.5 s after unpleasant and pleasant pictures, relative to controls. Importantly, unraveling how adaptive emotional recovery functions in the healthy population could serve as a useful comparison when recovery is compromised in clinical populations. A wealth of literature indeed demonstrates patients with depression and anxiety to ruminate and worry over past emotional events (for review Nolen-Hoeksema et al., 2008), which may be linked to the dysfunction of recovery mechanisms. For example, recovery from negative events in depressed patients may be stifled because of the sustainment of negative affect (Siegle et al., 2002), as well as the difficulty in maintaining positive affect (Heller et al., 2009).

One way to gage emotional recovery is through attentional paradigms. Attention and emotion have been shown to be strongly interconnected, with affective stimuli taking precedence over competing stimuli, regardless of task relevance (for review see Yiend, 2010). This effect has been well documented by means of an event-related potential (ERP) component known as the Late Positive Potential (LPP), which is located over centro-parietal sites at approximately 300 ms after stimulus onset, and is thought to reflect the process of sustained attention (Olofsson et al., 2008; Hajcak et al., 2010; Lang and Bradley, 2010). In emotional contexts, the LPP component is typically enhanced for arousing negative and positive pictures, relative to neutral, both during passive viewing (Cuthbert et al., 1999; Schupp et al., 2000), and concurrent task performance (Hajcak et al., 2007). This enlargement of the LPP for arousing stimuli, relative to neutral stimuli, has been postulated to signify the global inhibition of competing stimuli in the environment, permitting motivationally relevant stimuli to be selectively processed (Schupp et al., 2004; Brown et al., 2012). To test this notion, Schupp et al. (2004) presented emotional images in tandem with acoustic startle probes. Their results indicated negative and positive images to reliably inhibit the processing of secondary acoustic startle probes, denoted by larger LPPs to the arousing images and smaller P3 components to the probes. In addition, sustained attention as measured through the magnitude of the LPP has also been shown to extend beyond the offset of an emotional stimulus and to disrupt the processing of subsequent stimuli. For instance, the LPP has been shown to continue for up to 800 ms after pleasant pictures and for up to 1000 ms after unpleasant pictures (Hajcak and Olvet, 2008). Furthermore, Weinberg and Hajcak (2011) found pictures that elicited larger LPPs within individuals to predict slower reaction times (RTs) and reduced P300 amplitudes over parietal areas to subsequent categorization of shape targets. Given the temporality of attentional-emotional processes, represented through the LPP, these findings suggest that the LPP may serve as: (1) an important indicator of individual differences in the intensity of emotional reactivity, (2) a useful predictor of recovery outcomes e.g., a predictor of attentional interference on subsequent task-relevant stimuli.

Quantifying emotional recovery via the level of attentional modulation on task-relevant targets that appear after emotional stimuli may be useful in determining the lingering effect of emotion after offset. For instance, attentional interference or facilitation upon a following target can be considered a marker of continued processing of task-irrelevant emotional stimuli, with the former disrupting attention to following targets, whilst the latter widens attention to following targets. Furthermore, a recent body of behavioral research using rapid serial visual presentation tasks has provided ample evidence that viewing emotional stimuli can both interfere and facilitate the attentional processing of following targets, depending on the temporal proximity between stimuli (Bocanegra and Zeelenberg, 2009; Ciesielski et al., 2010). Bocanegra and Zeelenberg (2009) found emotional words impaired accuracy on subsequent neutral word targets when distances in time were as small as 50 ms and 500 ms, whilst longer time intervals of 1000 ms improved accuracy. Similarly, Ciesielski et al. (2010) observed that emotional picture distracters, particularly those exhibiting erotic and disgusting content, only reduced the participants' accuracy on a subsequent task during smaller distracter-target lags, e.g., 200 ms, 400 ms, and 600 ms. Longer lags, i.e., 800 ms, however, produced facilitation effects in accuracy. In addition, studies using target detection tasks have found comparable behavioral results. For example, Weinberg and Hajcak (2011) found interference, denoted as slower RTs on shape targets presented directly after emotional images (e.g., 0 ms).

Given the extent to which differential effects of attentional facilitation and interference appear dependent upon temporal aspects, as evidenced above, it can be postulated that several distinct mechanisms are at work during the recovery of an emotional stimulus. Indeed, support for this argument can be found from recent ERP studies, which demonstrate emotional pictures to modulate specific target ERP waveform components over time, thus indicating emotional stimuli to impact upon various stages of subsequent target processing (Ihssen et al., 2007; Weinberg and Hajcak, 2011; Brown et al., 2012). For example, Ihssen et al. (2007) found arousing images to disrupt processing of lexical targets as shown by slower RT and reduced amplitude on two ERP components: (1) the early attention-specific N1, observed over occipital sites, and time locked to 184–284 ms, and (2) the later LPP, observed over parieto-central regions and time locked to 412–712 ms. These effects occurred over three different temporal intervals between the emotional image and target, i.e., 80 ms, 200 ms, and 440 ms. Likewise, Brown et al. (2012) found that briefly presented negative images (e.g., 200 ms) disrupt processing on the early N1 to flashed probes, but not the N1 to Gabor patches, over short intervals of 570 ms between negative images and targets. Furthermore, Weinberg and Hajcak (2011) revealed emotional images to slow RTs and to attenuate subsequent P300 amplitude to shape targets which directly followed the images. The disparity between valence specificity in these studies may be due to differences in task type, specific state induced by the emotional images (positive vs. negative), as well as timing of the target stimuli presented. Despite this, it is important to note that these ERP studies are coherent in showing distraction from emotional stimuli on early ERP components locked to subsequent targets. In addition, the electrophysiological findings from these studies overlap with the behavioral research presented above, whereby shorter temporal proximities between an emotional prime and target result in interference effects. Yet, it remains unclear whether longer time intervals yield similar patterns of attentional interference or perhaps facilitation for electrophysiological and behavioral metrics.

In the study reported here, we used behavioral and ERP methodology in conjunction with an attentional paradigm to investigate: (1) the extent of recovery from arousing negative and positive stimuli, relative to neutral stimuli; (2) the impact of individual differences in emotional reactivity upon recovery speed. The experimental task consisted of presenting emotional images for 3 s, followed by a probe stimulus of 500 ms consisting of a neutral face-target controlled with FACSGen and validated in a previous study (Roesch et al., 2011). Participants were instructed to identify the gender of the face and respond accordingly. In addition, we manipulated the inter-stimulus interval (ISI) between the picture and face-target in the form of a fixation cross presented for a random period of time in two conditions, varying between 400–1500 ms and 2400–3500 ms, respectively. We used IAPS images (Lang et al., 2005) as emotional stimuli because they have been shown to induce emotion (Lang and Bradley, 2010), reliably modulate the LPP component (Olofsson et al., 2008; Hajcak et al., 2010) and impact subsequent task processing (Ihssen et al., 2007; Weinberg and Hajcak, 2011; Brown et al., 2012). Our subset of IAPS pictures consisted of negative and positive emotional pictures that were matched in arousal, as well as neutral pictures, to assess the influence of valence and arousal upon recovery outcomes. The LPP component to the emotional images was recorded, to serve as a metric of individual differences in emotional reactivity and a predictor of individual differences in emotional recovery, quantified as the level of interference on subsequent face-targets. Face stimuli were used as probes for a number of reasons. Firstly, we aimed to expand the line of behavioral research that had previously used categorization tasks to assess the extent of attentional capture by emotional stimuli after offset more generally (Ihssen et al., 2007) and within individuals (Weinberg and Hajcak, 2011). Secondly, we wanted to capture ERPs that have been shown to be modulated by attention, such as: (1) the face-specific N170, which is a negative potential occurring around 150–200 ms over occipito-temporal sites, and is thought to reflect early perceptual and holistic encoding (Bentin et al., 1996). (2) The P3 component, a positive deflection found over parieto-occipital areas around 300–400 ms, which has been associated with target detection (Schupp et al., 2004; Weinberg and Hajcak, 2011). Isolating those stages of processing that may show effects of preceding emotion-laden stimuli may be important for understanding mechanisms relevant to emotional reactivity and regulation. An advantage of using the FACSGen stimuli, compared to other face stimuli, is that the expressions are computer generated based upon parametrically controlled facial action units, which means that the expressions portrayed on our stimuli set are as intrinsically neutral as possible and are exactly the same across the set. Lastly, temporal intervals were included in experiment to examine how valence and arousal would impact the temporality of emotional recovery speed. We opted for shorter and longer temporal intervals because of the paucity of ERP research examining the impact of preceding emotion-laden stimuli on attention over a timescale of several seconds within groups and individuals.

Our main hypotheses were fourfold. Firstly, we expected negative and positive images to elicit more sustained attention than neutral images, indexed by larger LPP amplitudes for negative and positive images, relative to neutral (Lang and Bradley, 2010). Secondly, we expected arousing pictures to interfere with the subsequent processing of face-targets, as shown by slower RTs and smaller N170/P3 amplitudes on following face stimuli, compared to neutral pictures (Ihssen et al., 2007; Weinberg and Hajcak, 2011). Thirdly, modulation of RT and N170/P3 amplitude would be contingent upon the temporal interval between the arousing picture and target. We proposed that attentional interference between an arousing image and target will occur over shorter temporal intervals due to increased competition between the image and target, thus suggesting a slower recovery speed to emotional images, relative to neutral images (Bocanegra and Zeelenberg, 2009; Ciesielski et al., 2010). We expected this to be shown by slower RTs and smaller N170/P3 amplitudes to face-targets (Weinberg and Hajcak, 2011). Based on the behavioral findings of Bocanegra and Zeelenberg (2009) and Ciesielski et al. (2010), we predict that attentional facilitation will ensue when the temporal interval between an arousing picture and target is longer, as the competition between the image and target will be reduced but with a lingering effect of emotional image on attentional focus. We anticipated this to be evidenced by faster RTs and larger N170/P3 amplitudes to face-targets. Lastly, we examined how individual differences in emotional reactivity could predict speed of emotional recovery, by correlating IAPS-locked LPP values to RTs and N170/P3 amplitudes on subsequent early and late face-targets. We expected higher LPP values for arousing images to predict a more sustained impact on the face targets, reflected in larger differences between RTs and ERP amplitudes on following face-targets after arousing vs. neutral images. Given previous work showing interference by the LPP upon the visual P3 of subsequent targets specifically (see Weinberg and Hajcak, 2011), we predicted this relationship to be stronger for the P3 component than the N170.

## Materials and methods

### Participants

Twenty-five right-handed students from the University of Reading Psychology Department were recruited for this study (mean age = 20.2 years, 18 females and 7 males). All participants had normal or corrected to normal vision. Students provided written informed consent and received partial course credit for their participation. The procedure was approved by the University of Reading Ethics Committee.

### Stimuli

We selected 216 pictures from the IAPS (Lang et al., 2005), depicting seventy-two events from each valence category (see Table 1); negative, positive, and neutral. Mean (*SD*) normative ratings of valence across the negative pictures was 2.61 (1.57); for positive 7.41 (1.57); and for neutral, 5.00 (1.25). Mean valence ratings for each picture category were significantly different to the other picture categories, *p* < 0.001. For arousal, mean (*SD*) negative, and positive picture ratings were matched, negative 5.66 (2.22); positive 5.61 (2.28); neutral 3.20 (1.93). Negative and positive arousal ratings did not significantly differ, *p* = 0.6. Both negative and positive arousal ratings significantly differed from neutral, *p* < 0.001. The mean (*SD*) complexity and luminance of the images selected were matched across categories by using the scores of complexity and luminance (see also van Reekum et al., 2007): Complexity, negative 124606.40 (40955.07); positive 121928.66 (31956.27); neutral 126801.65 (45579.57), and luminance, negative 0.37 (0.13); positive 0.38 (0.13); neutral 0.37 (0.16). Mean complexity and luminance did not significantly differ across categories, *p* > 0.4.

**Table 1:** **Reference numbers to images taken from the International Affective Picture System (IAPS; Lang et al., 2005)**.

**Negative**		**Neutral**		**Positive**	
1052	9040	2038	2594	1463	7200
1111	9050	2102	2595	1710	7230
1220	9140	2104	2749	1722	7260
1274	9180	2191	2830	1811	7270
1301	9181	2210	2840	2058	7330
2095	9250	2214	4605	2071	7350
2141	9253	2215	5130	2150	7400
2683	9300	2235	5410	2160	7430
2688	9301	2271	5534	2208	7470
2691	9320	2272	5740	2209	7502
2710	9340	2280	5875	2216	7508
2751	9373	2305	7020	2224	8030
2981	9400	2357	7030	2340	8034
3015	9419	2381	7034	2345	8080
3051	9420	2383	7036	2346	8090
3061	9421	2385	7038	2352.1	8170
3160	9423	2393	7040	4599	8180
3181	9424	2396	7050	4603	8185
3215	9425	2397	7053	4610	8186
3220	9428	2440	7055	4623	8200
3230	9429	2441	7059	4626	8210
3350	9430	2446	7110	4640	8300
5971	9433	2480	7150	4641	8350
5973	9470	2485	7160	5260	8370
6213	9495	2491	7161	5270	8380
6242	9520	2493	7180	5450	8400
6243	9560	2506	7185	5470	8420
6244	9570	2512	7234	5480	8461
6540	9584	2513	7491	5600	8470
6570.1	9592	2514	7493	5621	8490
6571	9620	2515	7595	5623	8496
6821	9621	2516	7705	5629	8499
6830	9630	2518	7950	5700	8503
7359	9901	2570	8311	5830	8510
7380	9911	2579	9070	5833	8531
8485	9925	2593	9210	5910	8540

Pictures of thirty-six synthetic 3-dimensional neutral face stimuli (18 male and 18 female) were selected (see Table 2) from the stimuli used in the validation procedures for the FACSGen software (Roesch et al., 2011). As part of this validation procedure, 44 students from the University of Geneva were instructed to rate synthetic faces created with FaceGen Modeller (Singular Inversion Inc., 2012) on three continuous dimensions spanning 0–100: gender (anchored male-female), believability (anchored synthetic-believable), and intrinsic emotionality (anchored positive-neutral-negative). We selected thirty-six faces (18 male; 18 female) for being the most unambiguous gender-wise, the most believable, and the most emotionally neutral faces as possible. Mean (*SD*) normative ratings of gender for the seventy-two faces were; female 78.61 (5.895) and male 4.655 (3.46), where ratings of male and female gender significantly differed, *p* < 0.001. In addition, male and female faces were matched for mean (*SD*) ratings of neutral emotional expression and credibility of the face: Neutral expression, female 47.41 (6.46); male 48.16 (5.77), and credibility, female 52.21 (11.33); male 55.65 (9.6). Mean neutral expression or credibility ratings of male and female categories did not significantly differ, *p* > 0.2.

**Table 2:** **Reference numbers to faces taken from the Facial Action Coding System Generator (FACSGen: Roesch et al., 2011)**.

**Male**	**Female**
95	7
96	23
102	30
109	45
111	49
119	56
136	59
139	60
141	70
143	72
145	75
146	77
147	86
157	87
163	88
164	92
166	176
171	178

### Task design

All of the tasks were administered using E-Prime 2.0 (Psychology Software Tools Ltd, Pittsburgh, PA). Within each task, the experimental trials were randomized and the response button press on the mouse for the gender task was counterbalanced across participants. Tasks were presented on a Viewsonic 22 inch monitor with a 60 Hertz refresh rate. Screen resolution was set at 1024 × 768 pixels. For both the emotional recovery task and IAPS rating task, participants sat at approximately 60 cm from the screen. The resulting visual angles were: 19° × 15° for FACSGen faces and 35.6° × 22.5° for IAPS images.

#### Emotional recovery task

**Figure 1:**
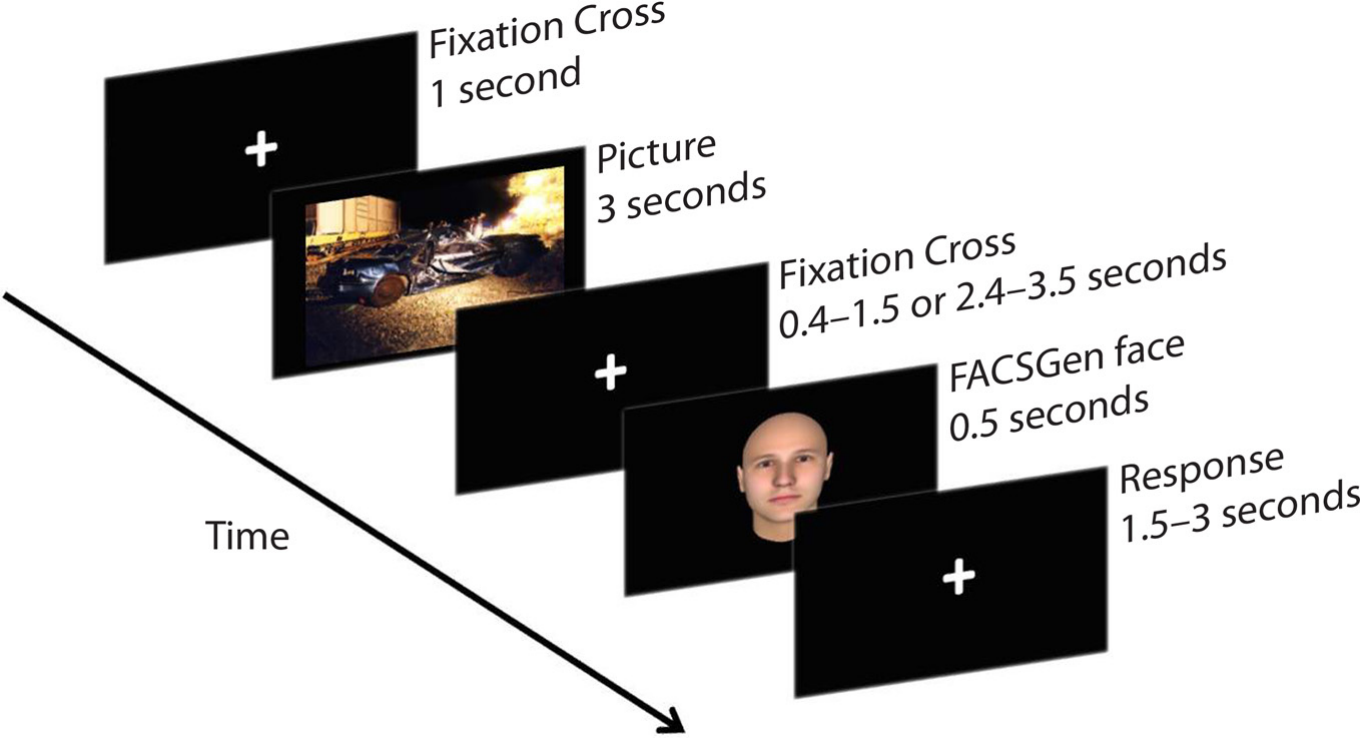
**A sample trial from the emotional recovery task: a fixation cross was presented at the center of the screen to direct participants' attention.** Next, an IAPS image was presented, followed by a variable temporal interval. Lastly, a face-target was briefly presented. Participants were instructed to watch each picture and identify the gender of the following face-target as quickly and as accurately as possible.

Participants were required to passively view emotional pictures and identify the gender of following neutral face-targets, by pressing the appropriate button on the mouse. Participants were instructed to focus on a fixation cross displayed between the picture and the face-target, to minimize noise from eye and muscle movement on the EEG. The face target was presented at a random time between either 400–1500 ms or 2400–3500 ms, in order to assess the temporality of emotional recovery, demonstrated as the degree of attentional spill-over from the previous emotional picture upon a subsequent face-target. The emotional recovery task consisted of 216 trials: 3 Valence (negative, neutral, positive) × 2 Temporal Interval (early: 400–1500 ms, late: 2400–3500 ms) × 36 Neutral Faces. Each IAPS picture was presented once; each neutral face was repeated six times and paired with a specific valence, which was counterbalanced across time and gender. A trial thus consisted of a 1000 ms fixation cross, 3000 ms IAPS picture presentation, 400–1500 ms or 2400–3500 ms fixation cross, 500 ms neutral face-target, and a 1500–3000 ms response window (see Figure 1).

#### IAPS rating task

Upon completion of the main experiment, all IAPS pictures were presented again to the participants, in a random order for 2 s. Participants were instructed to provide ratings on two dimensions, valence and arousal. The next trial started after participants completed both ratings using the keyboard. Each rating was provided on a nine point Likert scale, where participants were given instructions to rate valence, i.e., “how positive or negative you felt in response to the picture” and arousal, i.e., “the extent to which you felt calm or excited in response to the pictures.” For the valence ratings, a response of 9 represented “very pleasant” and 1 “very unpleasant,” while for arousal 1 represented “very calm” and 9 represented “very excited” (cf. Lang et al., 2005).

### Procedure

The current study was assessed and conducted in line with the ethics procedures in place at the University of Reading. Upon arrival, participants were informed of the experimental procedure and asked to complete a consent form. Participants were seated in a RF-shielded, sound-attenuated room for the EEG setup and experimental testing. Firstly, the participant's head was measured, in order to identify the correct size EEG cap. Secondly, once the cap was attached, the skin was cleaned with alcohol and a conductive gel was used to obtain sufficient impedance between the electrodes and scalp. When the EEG was stable and the participant was ready, the emotional recovery task was started on the computer. After completing the emotional recovery task, the EEG cap was removed. Next, participants performed the picture rating task. Lastly, participants were thanked for their participation and debriefed about the details of the study.

### Data collection and reduction

For EEG recording we used an MR compatible, 32-channel, powerpack operated amplifier (Brain Products, GmBH, Germany). Thirty-two Ag/AgCl pellet pin electrodes were positioned on an elastic cap according to the standard 10/20 system. There were three midline electrodes (Fz/Cz/Pz) and 14 electrodes over each hemisphere (FP1/FP2, F3/F4, F7/F8, FC1/FC2, FC5/FC6, FT9/FT10, C3/C4, CP1/CP2, CP5/CP6, T7/T8, TP9/TP10, P3/P4, P7/P8, and O1/O2). Electrode FCz served as the reference point and electrode AFz the ground. One electrode (IO) was placed on the outer canthi of the right eye for horizontal eye movement recordings. Electrode impedance was kept below 5 kΩ. EEG signals were recorded using Brain Vision Recorder Version 2.01 (Brain Products). Off-line EEG analysis was performed with Brain Vision Analyzer Version 2.01 (Brain Products). Firstly, the raw data were inspected for electromyographic (EMG) artifacts and other noise that could distort the EEG signal. These artifacts were highlighted and removed from the data. Secondly, data were filtered with a low cutoff of 0.1 Hz (Hajcak and Olvet, 2008) and a high cutoff of 40 Hz. Thirdly, to identify and remove eye movement artifacts, an ocular Independent Component Analysis was performed on the data. We used the IO electrode to identify horizontal eye movements and FP1 to identify vertical eye movements. Lastly, a semi-automated visual inspection for remaining physiological artifacts was made on each channel within each trial. Trials were rejected if there was: (1) a voltage step of more than 50 μV between sample points, (2) a voltage difference of 300 μV within a trial, and (3) a maximum voltage difference of less than 0.50 μV within 100 ms intervals (Hajcak and Olvet, 2008).

EEG data from the emotional pictures and face-targets were only segmented if the emotional picture and face-targets were followed by a correct response (95% of trials). After completion of these artifact rejection steps, 74% of emotional picture trials and 70% of face trials were included in the segmentation and averaging process. The percentage of picture trials across participants for each condition were: Negative Early = 75%; Negative Late = 71%; Neutral Early = 75%; Neutral Late = 74%; Positive Early = 75%; Positive Late = 71%. The percentage of face trials across participants for each condition were: Negative Early = 70%; Negative Late = 72%; Neutral Early = 71%; Neutral Late = 66%; Positive Early = 72%; Positive Late = 71%.

For emotional picture stimuli, segments were extracted from −200 ms before the image and 3000 ms after image onset. The remaining trials were −200 to 0 ms baseline corrected and averaged. The average voltage ^*^ ms from 300–1200 ms, 1200–2100 ms, and 2100–3000 ms post image onset was calculated for three electrodes associated with the LPP: Fz/Cz/Pz. The average voltage ^*^ ms values were then collapsed individually for Fz/Cz/Pz, for each subject and experimental condition.

Epochs around the face stimuli were extracted from −100 ms before the face-target and 600 ms after face-target onset. The remaining trials were then −100 to 0 ms baseline corrected and averaged. A peak detection method was used to locate the negative polarity of the N170 in TP9/TP10, and to identify the positive polarity of the P3 in P7/P8/Pz/O1/O2. The time parameters for finding the peaks were 140–220 ms for the N170 and 300–390 ms for the P3. Average amplitude values were then collapsed across each subject and condition: independently for TP9/TP10 electrodes for the N170 and across P7/P8/Pz/O1/O2 electrodes for the P3.

RTs in the emotional recovery task were scored for correct responses and only those RTs above 300 ms were retained (95% of trials). Accuracy scores from the emotional recovery task were expressed as the proportion of correct trials to total trials included.

The data of one participant was excluded from analyses due to loss of signal over the temporal electrode sites, thus leaving a total of 24 participants for statistical analyses.

## Results

### Main effects analysis

To test the extent to which IAPS stimuli modulate subsequent processing of neutral face information over time, we conducted a 3 Valence (negative, neutral, positive) × 2 Temporal Interval (early: 400–1500 ms, late: 2400–3500 ms) repeated measures analysis of variance (ANOVA) for RT, accuracy, and P3 amplitude. In addition, a 3 Valence (negative, neutral, positive) × 2 Temporal Interval (early: 400–1500 ms, late: 2400–3500 ms) × 2 Hemisphere (right, left) repeated measures ANOVA was conducted on N170 amplitude. The additional factor of hemisphere was included to assess the laterality of the N170, where N170 amplitudes are found to be typically larger over the right hemisphere sites, compared to left (Bentin et al., 1996). To examine LPP amplitude, we utilized a 3 Valence (negative, neutral, positive) × 2 Temporal Interval (early: 400–1500 ms, late: 2400–3500 ms) × 3 LPP Window (early: 300–1200 ms, middle: 1200–2100 ms, late: 2100–3000 ms) × 3 Lead (Fz, Cz, Pz) repeated measures ANOVA. Furthermore, to assess whether our participants' ratings of IAPS reflected the normative ratings, we used a 3 Picture (negative, neutral, positive) repeated ANOVA for IAPS ratings. Significant predicted effects in the omnibus tests were followed up with pairwise comparisons. All analyses were conducted using SPSS 17.0 (IBM Ltd).

#### IAPS ratings

**Figure 2:**
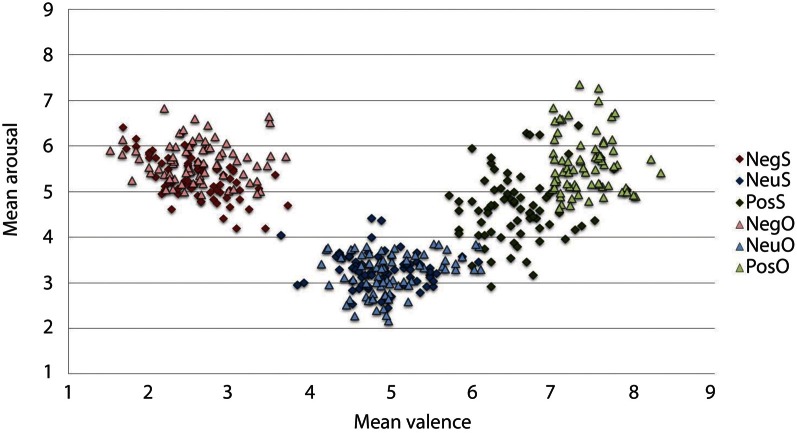
**Mean IAPS valence and arousal ratings from the current study sample and the original sample for the 216 pictures selected.** On the valence scale, lower numbers denote negative valence, whilst higher numbers reflect positive valence. Similarly, in the arousal scale, high arousal is reflected by higher figures and lower arousal by lower figures. As expected, the current sample rated negative images as high arousing and unpleasant, and neutral images as low in arousal and neither pleasant or unpleasant. Positive images, however, have more variation in arousal ratings, particularly when comparing them to the original sample. The current experimental sample ratings may be typical for a young British sample, given that the images selected were controlled for arousal and valence based upon the original ratings that came with IAPS set (Lang et al., 2005). Neg, Negative; Neu, Neutral; Pos, Positive; S, current study sample ratings; O, original IAPS sample ratings.

The IAPS ratings produced a significant main effect of Valence, *F*_(2, 46)_ = 156.664, *p* < 0.001. Participants reported negative pictures (*M* = 2.52, *SD* = 0.88) to be the most unpleasant, positive pictures (*M* = 6.50, *SD* = 0.81) as the most pleasant and neutral ratings (*M* = 4.83, *SD* = 0.75) as neither unpleasant nor pleasant, *p* < 0.001. The ANOVA revealed arousal ratings to have a main effect of Valence, *F*_(2, 46)_ = 2.545, *p* < 0.001. Both negative (*M* = 5.31, *SD* = 1.58) and positive (*M* = 4.66, *SD* = 1.75) arousal ratings significantly differed from neutral (*M* = 3.29, *SD* = 1.50), *p* < 0.001. Although the arousal ratings were higher for negative than positive pictures in our sample, negative arousal ratings were not significantly different from arousal ratings of positive pictures, *p* = 0.087. The spread of arousal ratings of the positive pictures was higher in our sample relative to the normative ratings (see Figure 2).

#### IAPS-elicited LPP

**Figure 3:**
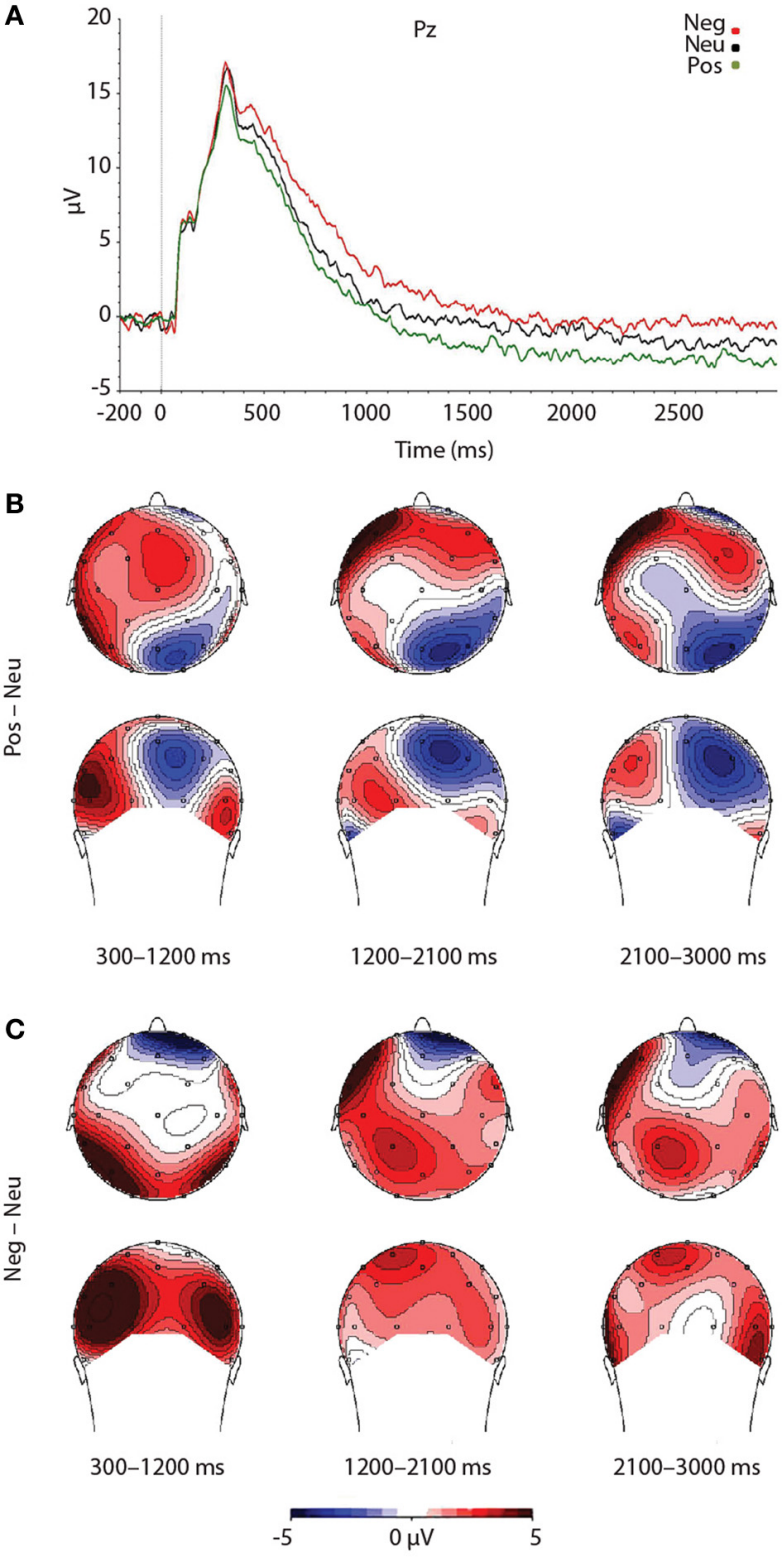
**Figure demonstrating: **(A)** Grand-averaged ERP waveforms of subjects during emotional picture presentation for each valence.** The Pz sensor was selected for representing the LPP component over parietal regions, where the LPP was maximal. **(B)** Voltage difference maps across the LPP for negative images, relative to neutral images. **(C)** Voltage difference maps across the LPP for positive images, relative to neutral images. Neg, Negative; Neu, Neutral; Pos, Positive; μV, microvolts; ms, milliseconds.

As expected, a significant main effect of Valence was found, *F*_(2, 46)_ = 7.492, *p* = 0.002. Findings were partially in line with predictions, as LPP amplitudes were larger for negative pictures, relative to neutral pictures, at trend level, *p* = 0.056 (see Figure 3). Reflecting the (non-significant) effect observed in the IAPS ratings, negative pictures evoked larger LPPs than positive pictures, *p* < 0.001. Moreover, the LPP amplitude to positive pictures and neutral pictures did not significantly differ, *p* = 0.163 (see Figure 3 and Table 3).

**Table 3:** **Summary of means (SD) for each dependent measure as a function of picture valence, separately for early and late temporal interval face-targets**.

**Measure**	**Early**			**Late**		
	**Negative**	**Neutral**	**Positive**	**Negative**	**Neutral**	**Positive**
**BEHAVIORAL**						
RT (ms)	666.06 (139.98)	668.84 (138.55)	667.24 (150.35)	631.73 (128.06)	647.42 (131.51)	641.09 (120.62)
ACC	0.94 (0.04)	0.96 (0.03)	0.95 (0.04)	0.96 (0.04)	0.95 (0.05)	0.95 (0.05)
**FACE-LOCKED ERPs**						
N170 (μV)	−8.36 (5.21)	−9.35 (5.24)	−8.74 (5.45)	−11.10 (5.81)	-12.03 (5.97)	−11.44 (5.70)
P3 (μV)	19.26 (5.55)	17.46 (5.64)	18.99 (6.49)	15.99 (6.98)	14.89 (7.31)	16.07 (8.00)
**IAPS-LOCKED ERPs**						
LPP (μV ^*^ ms)	2843.84 (4114.14)	1611.45 (3200.67)	535.69 (2636.49)	3259.33 (3327.15)	1808.66 (3186.92)	1248.49 (3779.14)

IAPS, International Affective Picture System; ERPs, Event-related potentials; N170 and P3 amplitude measured in microvolts; LPP, late positive potential amplitude measured in microvolts ^*^ milliseconds; RT, reaction time measured in milliseconds; ACC, proportion accuracy score.

To test that the LPP waveforms' temporality and topography was comparable to previous research (e.g., Olofsson et al., 2008; Hajcak et al., 2010; Lang and Bradley, 2010) we split the LPP into three windows and assessed the LPP at each lead. As expected, the analysis yielded significant interactions between Lead × Valence × LPP Window, *F*_(8, 184)_ = 2.059, *p* = 0.042, Lead × Valence, *F*_(4, 92)_ = 12.235, *p* < 0.001, and Lead × LPP Window, *F*_(4, 92)_ = 124.989, *p* < 0.001. The results were in accordance with previous studies that have examined the LPP (see Figure 3 and Table 4), as negative images had the largest centro-parietal activation and the smallest frontal activation during the early portion of the LPP, compared to neutral and positive images. In addition, within the middle portion of the LPP, negative images were found to elicit the strongest activity in central areas, relative to positive and neutral images. Furthermore, in the late portion of the LPP, negative images elicited more activity in centro-parietal regions than neutral and positive images. The LPP for neutral images were larger than positive images during middle and late windows, but only over parietal regions. The ANOVA also revealed a main effect of Lead, *F*_(2, 46)_ = 4.897, *p* < 0.012, and Window, *F*_(2, 46)_ = 115.590, *p* < 0.001, where the LPP was maximal: (1) at the Pz electrode, followed by Cz, and Fz electrodes. (2) In the early window, followed by the middle and late windows. No significant interaction between Valence × LPP Window was found, *F*_(4, 92)_ = 0.387, *p* = 0.817.

**Table 4:** **Summary of means (SD) for the LPP as a function of picture valence, window, and lead**.

IAPS-locked LPP at each lead (μV ^*^ ms)	**Early**			**Middle**			**Late**		
	**Negative**	**Neutral**	**Positive**	**Negative**	**Neutral**	**Positive**	**Negative**	**Neutral**	**Positive**
Fz	−1855.85_a_ (3346.85)	−770.30_b_ (2883.90)	−904.33_bc_ (3129.23)	−791.99_a_ (2825.47)	−56.04_ab_ (2436.742)	834.71_b_ (2082.33)	−297.19_a_ (3232.13)	231.76_ab_ (2595.35)	1067.98_b_ (2442.37)
Cz	4089.91_a_ (2036.926)	3032.69_b_ (2013.362)	2874.85_bc_ (1808.24)	1694.53_s_ (2307.81)	519.18_b_ (2071.53)	110.99_b_ (1918.96)	1396.79_a_ (2632.48)	−159.69_b_ (2259.08)	−527.82_b_ (2305.09)
Pz	12829.49_a_ (6865.04)	10142.60_b_ (5783.34)	8563.41_bc_ (6241.78)	1285.88_a_ (6958.60)	−474.70_a_ (4963.65)	−2729.79_b_ (5546.25)	−42.03_a_ (6284.98)	−2205.13_ab_ (4582.51)	−3937.41_b_ (5262.74)

IAPS, International Affective Picture System; LPP, late positive potential amplitude measured in microvolts ^*^ milliseconds. Means that do not share subscripts within rows from the same window condition are significantly different at the p < 0.05 based on Fisher's LSD post-hoc paired comparisons.

As a control, we tested for random differences in LPP values for the pictures preceding early vs. late face-targets. There was no Valence × Temporal Interval interaction, *F*_(2, 46)_ = 0.192, *p* = 0.826, for the LPP. Thus, while negative pictures elicited a higher LPP than positive and neutral pictures, no significant difference between LPP values from valence × early vs. late face conditions was found (see Table 3). No other interaction effects with Time were found, largest *F* = 2.18, *n.s*.

#### RT

While the average RT to faces following negative pictures were faster than those following positive and neutral pictures in the late interval, we found no significant main effect of Valence for RT, *F*_(2, 46)_ = 0.974, *p* = 0.385, nor Valence × Temporal Interval for RT, *F*_(2, 46)_ = 0.331, *p* = 0.720. As anticipated, however, RT was faster for later face-targets, compared to earlier face-targets, as reflected in a main effect of Temporal Interval, *F*_(1, 23)_ = 43.250, *p* < 0.001 (see Table 3).

#### Accuracy

Accuracy scores in emotional recovery task were relatively high across conditions, with on average 95% correct responses (see Table 3). Picture valence did not impact target-face Accuracy scores, *F*_(2, 46)_ = 0.247, *p* = 0.782, nor was there an effect of Valence × Temporal Interval, *F*_(2, 46)_ = 1.369, *p* = 0.265 or main effect of Temporal Interval, *F*_(1, 23)_ = 0.919, *p* = 0.348.

#### The face-locked N170 component

The N170 amplitudes revealed no main effect of Valence, *F*_(2, 46)_ = 1.444, *p* = 0.247, or Valence × Temporal Interval interaction, *F*_(2, 46)_ = 0.002, *p* = 0.998. Reflecting the behavioral effect observed for RT, the results yielded a significant main effect of Temporal Interval on N170 amplitude, *F*_(1, 23)_ = 24.684, *p* < 0.001 (see Table 3), whereby N170 amplitudes were potentiated for the late face-targets, relative to the early face-targets, *p* < 0.001. While larger N170 amplitudes were exhibited on the right TP10 electrode (*M* = −10.46 μV, *SD* = 5.43 μV), relative to the left TP9 electrode (*M* = −9.88, *SD* = 5.10 μV), this difference was not significant, *F*_(1, 23)_ = 0.638, *p* = 0.433. No other interaction effects with Hemisphere were found, largest *F* = 1.21, *n.s*.

#### The face-locked P3 component

**Figure 4:**
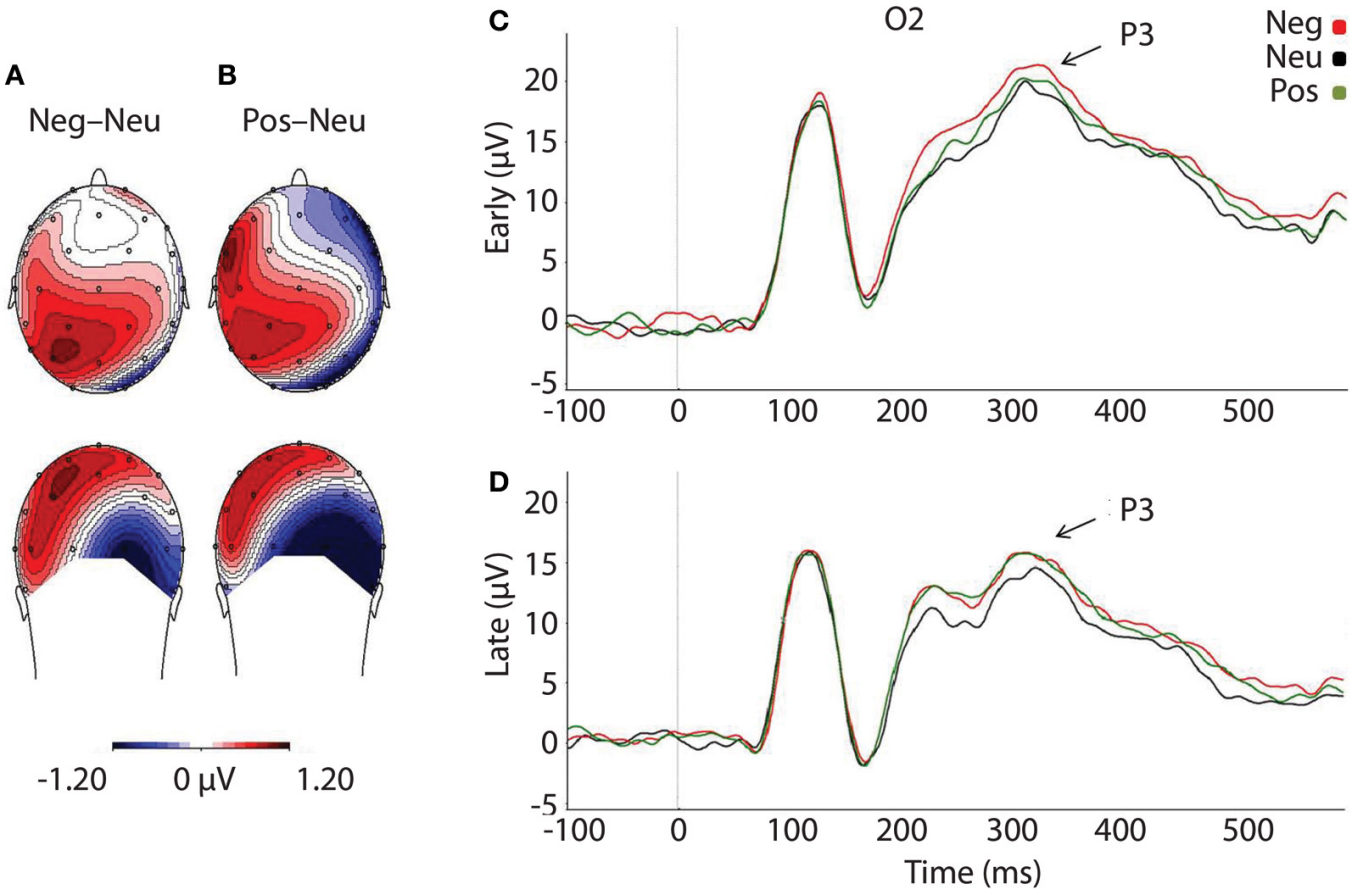
**Illustration depicting: **(A)** Voltage difference map for the P3 component (350 ms) when faces followed negative images, relative to neutral. (B)** Voltage difference map for the P3 component (350 ms) when positive images preceded faces, relative to neutral images. **(C)** Grand-averaged ERP waveforms of subjects during face-targets in each valence by early temporal interval condition **(D)** Grand-averaged ERP waveforms of subjects during face-targets in each valence by late temporal interval condition. The O2 sensor was selected for representing the P3 component over the right occipital hemisphere, where the P3 was maximal. Neg, Negative; Neu, Neutral; Pos, Positive; μ V, microvolts; ms, milliseconds.

As predicted, P3 amplitudes revealed a main effect of Valence, *F*_(2, 46)_ = 4.024, *p* = 0.025, where P3 amplitudes were accentuated for faces that had followed negative, *p* = 0.012, and positive pictures, *p* = 0.010, relative to neutral (see Table 3 and Figure 4). However, there was no significant difference in P3 amplitude between faces that had followed negative vs. positive pictures, *p* = 0.890. We did not find support for the prediction that the impact of picture valence on the face-locked P3 would dissipate over time, however; Valence × Temporal Interval, *F*_(2, 46)_ = 0.224, *p* = 0.800. Similar to N170, the ANOVA revealed a significant main effect of Temporal Interval on P3 amplitude, *F*_(1, 23)_ = 17.384, *p* < 0.001, where P3 amplitudes were larger for early face-targets, relative to late face-targets, *p* < 0.001. The same analyses, ran on the area metric data instead of the amplitudes at peak, yielded comparable results: Main effect of Valence, *F*_(2, 46)_ = 4.749, *p* = 0.013; and individual differences in the negative late—neutral late condition, *r* = −0.498, *p* = 0.013.

### Individual differences analysis

To investigate what impact the reactivity to emotional pictures had upon an individuals' recovery speed, we correlated difference scores for the LPP and P3 components in negative and positive conditions, relative to neutral, for both temporal interval conditions. We considered correlations to be significant if the *p*-value was lower than 0.0125 (i.e., *p* < 0.05, Bonferroni corrected for the four conditions of interest: negative early—neutral early, positive early—neutral early, negative late—neutral late, and positive late—neutral late). Temporal specificity was assessed by conducting a test of significant difference between the two correlation coefficients, if a significant correlation was found for one temporal interval condition but not the other (e.g., for negative early—neutral early but not for negative late—neutral late).

We focused our individual difference analyses on the LPP and P3 because both measures yielded a main effect of Valence in the ANOVA. Nevertheless, we conducted the same correlations between the LPP and RT/N170 as a control, despite finding no main effect of Valence in these measures. For the individual difference analysis, we collapsed the LPP across windows (300–3000 ms) and centro-parietal (Cz, Pz) electrodes, in order to increase predictive power, given that only subtle changes in Cz and Pz electrodes were found when the LPP was split into windows, and that the Fz electrode showed little valence modulation.

#### Relationship between reactivity and recovery

**Figure 5:**
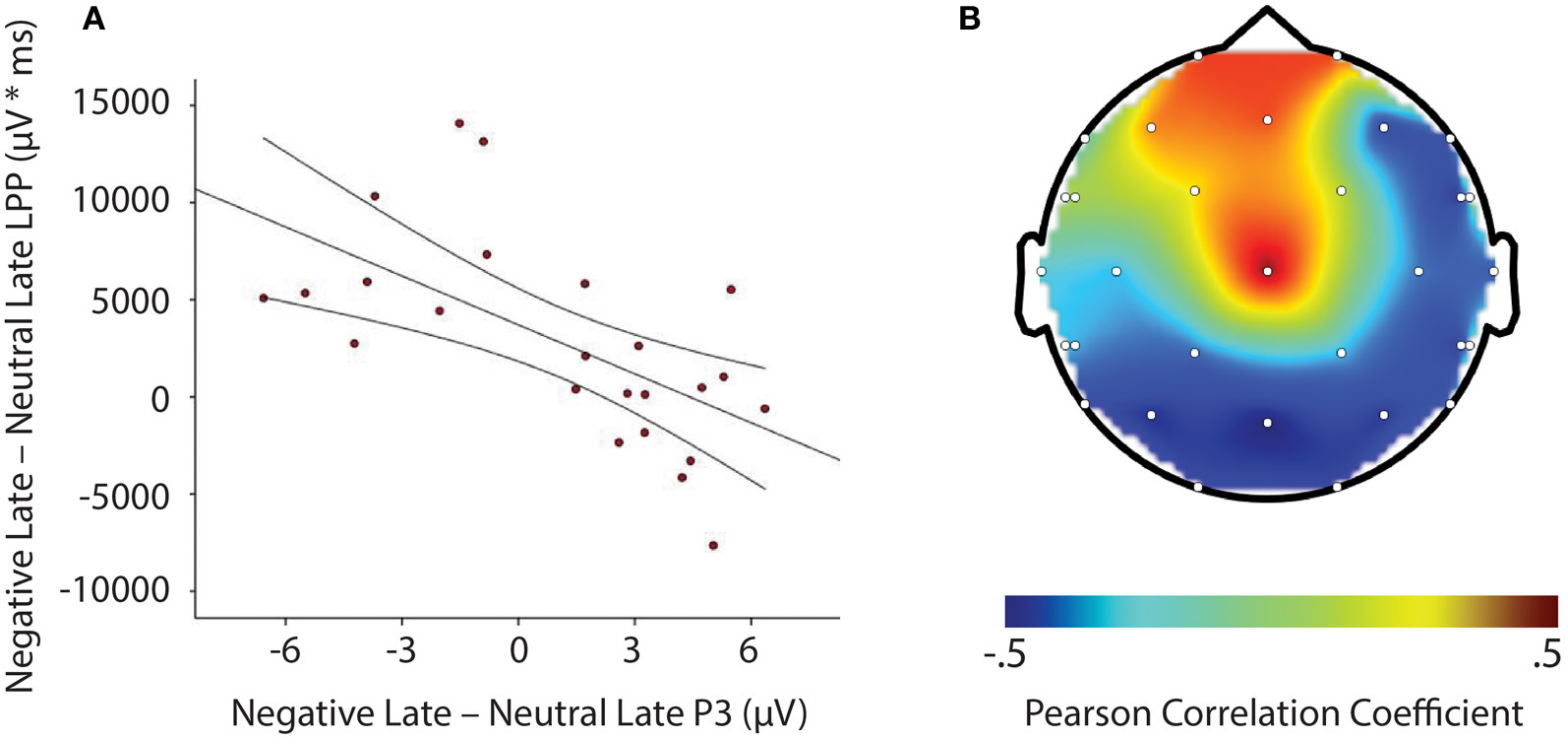
Illustration demonstrating: **(A)** Correlation of difference scores from negative early minus neutral late conditions for P3 amplitude and LPP amplitude ^*^ time. (B) A topographic representation of correlations between P3 amplitude and LPP difference scores from negative early minus neutral late conditions across the entire scalp. Larger LPP amplitudes to negative pictures induce smaller P3 amplitudes to following face-targets that are presented later in time, thus suggesting reactivity to predict recovery outcomes toward an emotional picture stimulus. μV, microvolts; μV ^*^ ms, microvolts by milliseconds.

**Figure 6:**
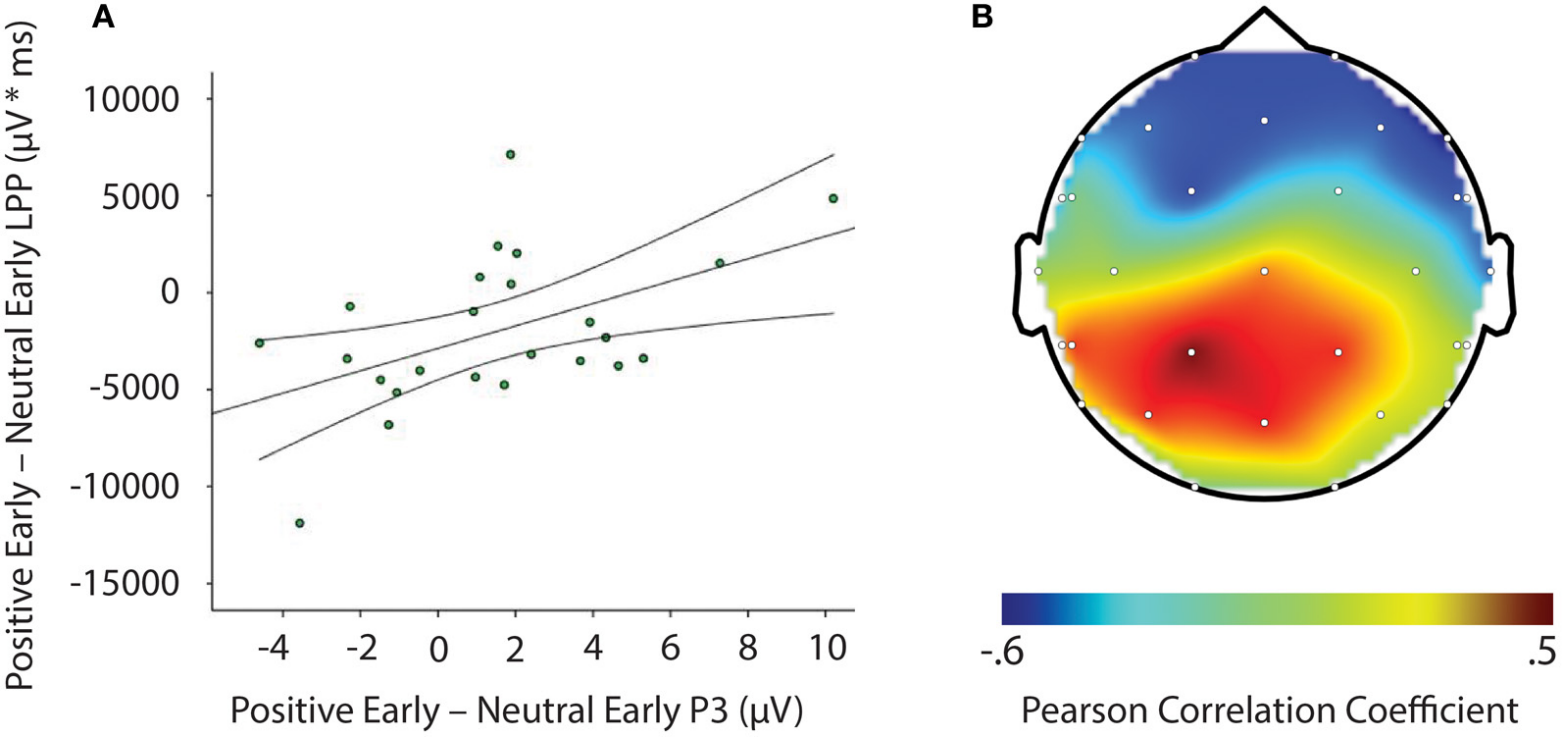
Image signifying: **(A)** Correlation of difference scores from positive early minus neutral early conditions for P3 amplitude and LPP amplitude ^*^ time. (B) A topographic representation of correlations between P3 amplitude and LPP difference scores from positive early minus neutral early conditions across the entire scalp. Larger LPP amplitudes to positive pictures generate enahnced P3 amplitudes to following face-targets that are presented earlier in time. These results suggest reactivity to predict recovery outcomes toward an emotional picture stimulus. μV, microvolts; μV ^*^ ms, microvolts by milliseconds.

LPP amplitude significantly correlated with P3 amplitude in the negative late—neutral late comparison, *r* = −0.611, *p* = 0.002, thus demonstrating individuals who had larger LPP amplitudes to negative pictures to show reduced P3 facilitation, even interference (i.e., a negative difference score) P3 amplitude on later face-targets presented after these negative images, relative to neutral images (see Figure 5). Additionally, temporal interval specificity was found for the negative—neutral LPP-P3 relationship, whereby the two correlation coefficients of negative early—neutral early (*r* = −0.017, *p* = 0.937) and negative late—neutral late significantly differed, *p* = 0.0069. Furthermore, we also found LPP amplitude to significantly correlate with P3 amplitude in the positive early—neutral early comparison, *r* = 0.503, *p* = 0.012. Individuals with larger LPP amplitudes to positive pictures showed an enhanced P3, denoting facilitation (i.e., a positive difference score) on earlier face-targets presented after these positive images, relative to neutral images (see Figure 6). This effect was temporally specific for the positive-neutral LPP-P3 relationship, as we found the two correlation coefficients of positive early—neutral early and positive late—neutral late (*r* = −0.216, *p* = 0.310) to significantly differ, *p* = 0.0037. However, the LPP did not predict any other dependent measures of recovery, such as RT or N170 (correlations did not survive Bonferonni correction).

## Discussion

In the current study, we found negative pictures to consistently evoke larger LPPs, relative to positive and neutral pictures. Both negative and positive images facilitated target detection of following face-targets, as indexed by increased face-locked P3 amplitudes. This relationship was found for both temporal interval delays between emotional image offset and face-target onset. However, individual differences in emotional reactivity to negative images and positive images, as demonstrated by the LPP, predicted the extent of interference and facilitation on subsequent face-targets after image offset: (1) The stronger the LPP to a negative image the smaller the P3 amplitude to a subsequent late face-target, thus suggesting a slower emotional recovery in those individuals who responded more strongly to the negative information. (2) The stronger the LPP to a positive image, the larger the P3 amplitude to a subsequent early face-target, therefore indicating a (short-lived) widening of attention in those individuals who responded more strongly to the positive information. No other metrics of emotional recovery (e.g., RT and the N170 component) were shown to be modulated by preceding emotional pictures at the group or individual difference level.

Our findings suggest preceding negative and positive stimuli to reliably modulate attentional processes, as indexed in our study by subsequent face-locked P3 amplitude, similarly to other ERP components (Ihssen et al., 2007; Weinberg and Hajcak, 2011; Brown et al., 2012) and other metrics of attention such as defense startle reflex (Jackson et al., 2003; Larson et al., 2007). Such findings are in line with past behavioral experiments which suggest emotional stimuli to increase attentional vigilance toward following task-relevant stimuli, when the temporal intervals between stimuli are longer (Bocanegra and Zeelenberg, 2009; Ciesielski et al., 2010). The main effect of valence was in part not in the predicted direction however, as we expected interference in the early time window, denoted as smaller P3 amplitudes after a negative and positive picture, similar to that reported by Ihssen et al. (2007). In addition, we did not find a valence by temporal interval interaction on P3 amplitude. These findings may reflect the current study design, as we incorporated longer temporal intervals between emotional pictures and face-targets compared to other studies, which have used either immediate presentation (Weinberg and Hajcak, 2011) or shorter temporal intervals (Ihssen et al., 2007; Bocanegra and Zeelenberg, 2009; Ciesielski et al., 2010).

Importantly, we found the P3 component to exhibit facilitation, or even interference effects based on valence and temporal interval in the individual difference data, as: (1) larger LPPs to negative relative to neutral pictures significantly predicted smaller P3 amplitudes to faces that were presented later in time, and (2) larger LPPs to positive relative to neutral pictures significantly predicted larger P3 amplitudes to faces that were presented earlier in time. Such effects suggest heightened reactivity to negative and positive stimuli to disrupt or facilitate the processing of following face-targets, dependent on the temporal interval between emotional stimuli and subsequent face-targets. These P3 results, particularly from the negative late condition, are comparable to individual difference findings by Weinberg and Hajcak (2011), who demonstrated that individuals with larger LPPs to emotional images to have smaller parietal P300 amplitude on shape targets that were to be categorized. In contrast, however, the individual differences in this study were dependent on the temporal specificity between the emotional pictures and face-targets. The difference in findings may be due to the current study samples interpretation of the IAPS content (see Weinberg and Hajcak, 2010) as we found negative pictures to elicit the largest LPP and highest arousal ratings, whilst we found positive images to produce a small LPP and lower arousal ratings than negative images. Nevertheless, the results from this study are novel and highlight the importance of individual differences in temporal dynamics of attention toward emotional events. More specifically, those individuals who allocated more attention to: (1) negative, relative to neutral images showed interference from negative images upon face-targets up to 2400–3500 ms after negative image onset, and (2) positive, relative to neutral images demonstrated facilitation from positive images upon face-targets up to 400–1500 ms after positive image offset. With the current data set, it can be posited that greater sustained attention to affective stimuli may lead to continued processing after offset of the affective stimulus (Davidson, 1998; Hajcak and Olvet, 2008). But what that continued processing consists of is hitherto not clear. Tentatively, we can postulate that it may involve further processing of the emotional events' content and relevance. Understanding this continued processing of emotional stimuli may be vital for identifying maladaptive recovery in affective disorders due to e.g., extreme worry and rumination in anxiety and depression (Siegle et al., 2002; Larson et al., 2007; Nolen-Hoeksema et al., 2008; Heller et al., 2009).

We did not find RT or the N170 component to vary as a function of valence or valence by temporal interval interaction. These observations indicate that early perceptual encoding of faces may not be modulated by previous attentional engagement with valenced information. However, other reports in the literature have also demonstrated mixed results on the influence of emotional picture stimuli on perceptual bottom up processing. For instance, emotional stimuli have been shown to disrupt early ERP components on flash probe tasks but not Gabor tasks (Brown et al., 2012). The N170 results found here may also reflect the longer temporal interval manipulations. More specifically, earlier ERP components have been found to be modulated when the temporal intervals between stimuli are relatively short (Ihssen et al., 2007; Brown et al., 2012). With regards to RT data, we postulate that a combination of task factors may have masked possible attentional effects. Firstly, the demands of the current task may have not been particularly challenging (Brown et al., 2012), as demonstrated by the high accuracy scores found. Secondly, in our task there was large visual disparity between the emotional stimuli and face-targets, whilst previous studies have used the same stimuli for affective primes and task-relevant targets e.g., words (Bocanegra and Zeelenberg, 2009; but see Ihssen et al., 2007). Thirdly, the task used longer temporal distances between emotional pictures and face-targets, whilst in contrast previous studies have enlisted immediate presentation (Weinberg and Hajcak, 2011) or shorter temporal intervals (Ihssen et al., 2007; Bocanegra and Zeelenberg, 2009; Ciesielski et al., 2010) between emotional distractor and neutral target presentation. Given these points, the gender categorization task utilized may have been a less sensitive probe for emotional recovery, compared to previous studies (e.g., Ihssen et al., 2007; Bocanegra and Zeelenberg, 2009; Ciesielski et al., 2010; Weinberg and Hajcak, 2011). Future research should aim to develop more sensitive task designs, in order to elucidate emotional recovery mechanisms.

Results revealed a main effect of temporal interval, where RTs were shown to be faster and face-locked N170 amplitudes were enhanced when faces were presented in the later temporal interval (e.g., 2400–3500 ms), relative to the earlier temporal interval (e.g., 400–1500 ms). Face-locked P3 amplitudes, however, were shown to be larger in earlier time intervals, relative to later time intervals, indicating a reverse effect to that of the N170. We propose the differential modulation of temporal interval found upon the N170 and P3 to reflect a perceptual enhancement trade-off, where: (1) the N170 to early face-targets is reduced because of the competition between the previous attention demanding stimulus and face-target, thus resulting in a larger P3 to compensate for this detriment, (2) the N170 to later face-targets is enhanced due to less competition between the previous attention demanding stimulus and face-target, which subsequently allows for re-orienting of attention and anticipation of face-target onset, and therefore this consequently reduces the size of the P3 component. Overall, these behavioral and electrophysiological findings are in line with temporal attention research (for review see Correa et al., 2006), as previous work has shown both early and late ERP components to be reliably modulated by temporal expectations in this fashion when the task at hand is perceptually demanding. These findings indicate the experimental task to be sufficiently robust in producing perceptual effects.

In conclusion, the present study demonstrates emotional events to modulate subsequent processing on face-targets, indexed by the P3 component, but not early perceptual processes on face-targets, indexed by the N170 component. Both negative and positive pictures enhanced P3 amplitude on subsequent face-targets, regardless of whether the temporal interval was early or late between picture and target e.g., 400–1500 ms and 2400–3500 ms. These results indicate emotional stimuli to accentuate attentional processing on subsequent face-targets even during longer temporal intervals (Bocanegra and Zeelenberg, 2009; Ciesielski et al., 2010). At the individual level, larger LPP magnitude to negative relative to neutral images was found to predict smaller P3 amplitudes on following face-targets uniquely in the later temporal interval, i.e., 2400–3500 ms, whereas larger LPP magnitude to positive relative to neutral images was found to predict enhanced P3 amplitudes on following face-targets exclusively in the earlier temporal interval, i.e., 400–1500 ms. That is, in the face of overall facilitation, those individuals who responded more strongly to negative stimuli produced attentional interference from the negative stimuli in the late stages of recovery, whilst those individuals who reacted more strongly to positive stimuli produced attentional facilitation from the positive stimuli in the earlier stages of recovery. Overall, these findings confirm the LPP to serve as a useful metric of emotional reactivity (Lang and Bradley, 2010), as well as a useful predictor of emotional recovery (Weinberg and Hajcak, 2011). In addition, the face-locked P3 component can be used as a marker to assess the extent of emotional recovery, similar to that of other measures which show emotion modulation after stimulus offset, such as the N1 (Ihssen et al., 2007; Brown et al., 2012), P300 (Weinberg and Hajcak, 2011) and defense startle reflex (Jackson et al., 2003; Larson et al., 2007). Further work using attentional paradigms in combination with ERP methodology is needed in order to further specify the role of individual differences in emotional reactivity upon attention and emotional recovery. Isolating those psychological processes that are relevant to adaptive emotional recovery may provide important information for researchers aiming to improve health and well-being in those populations where emotional recovery is compromised.

### Conflict of interest statement

The authors declare that the research was conducted in the absence of any commercial or financial relationships that could be construed as a potential conflict of interest.
